# Seroprevalence and associated risk factors of peste des petits ruminants among ovine and caprine in selected districts of Afar region, Ethiopia

**DOI:** 10.1186/s12917-022-03528-6

**Published:** 2022-12-09

**Authors:** Teshager Dubie, Betelhem Dagnew, Esrael Gelo, Wossene Negash, Fentaw Hussein, Mulatu Woldehana

**Affiliations:** College of Veterinary Medicine, Samara University, P.O. Box 132, Samara, Ethiopia

**Keywords:** Afar, Associated risk factors, C-ELISA, PPRV, Seroprevalence, Small ruminants

## Abstract

**Background:**

A Peste des petits ruminant is an acute, highly contagious and economically important transboundary viral disease of small ruminants. Despite the fact that food and agriculture organization and world organization for animal health plan to eradicate the disease by 2030, some studies indicated an increasing seropositivity of PPR infection in sheep and goats in Ethiopia. A cross-sectional study was employed to estimate the seroprevalence of PPR and to assess risk factors during the study period, February to April, 2020. Following purposive selection of the study districts, simple random sampling technique was employed to select individual animal during sample collection. A total of 384 serum samples were collected from apparently healthy sheep and goats. Competitive Enzyme Linked Immunosorbent Assay was used to detect the presence of antibodies against PPR at national veterinary institute. Descriptive statistics, Pearson’s chi-square (X^2^) and logistic regression analysis were used is this study.

**Results:**

The overall animal level seroprevalence of PPR virus was found to be 60.15% (*n* = 231/384) and species level prevalence rate was found to be 38.18% (*n* = 42) in sheep and 68.98% (*n* = 189) in goats in the study areas. Among the associated risk factors considered; species, sex, age and herd sizes were significantly associated (*P* < 0.05) with the disease occurrence. Among the associated risk factors considered in this study, species, sex, age and herd size were found to be statistically associated with the seropositivity of PPR infection.

**Conclusion:**

The present study finding revealed that a higher seroprevalence of PPR virus infection and this confirms peste des petits ruminant virus is circulating in Afar region. Further studies should be carried out on the entire region to determine PPR seroprevalence and to develop appropriate control and eradication strategies of PPR disease.

## Background

Ethiopia is the first in Africa in terms of livestock population, with an estimated 65.35 million cattle, 39.89 million sheep, 50.50 million goats, and 48.96 million chickens [1]. Small ruminants are important asset of livestock keepers as owing to their faster growth rates, require small investments, have high fertility (shorter production cycles) and better adaptability even in harsh environments as compared to large ruminants [2, 3]. In spite of having such huge small ruminant resources, the country could not utilize the sector as a result of extremely rampant livestock diseases [4, 5]. Among those infectious diseases of small ruminants, peste des petits ruminants (PPR), sheep pox (SPP) and goat pox (GTP) are major problems of small ruminants and widely distributed in all regions of the country [6].

Peste des petits ruminant (PPR) is an acute, highly contagious, remarkable and economically important transboundary viral disease of small ruminants, which is listed by world organization for animal health (OIE) as notifiable disease [7, 8]. The disease is caused by PPR virus (PPRV), RNA virus belongs to order Mononegavirales, the a member of genus morbillivirus of family Paramyxoviridae [9]. Peste des petits ruminant (PPR) is the next priority of small ruminants disease targeted for global eradication campaign by Food and Agricultural Organization (FAO) and the World Organization for Animal Health (OIE) [10, 11]. Peste des Petits Ruminants (PPR) is a rinderpest-like disease of goats and sheep having many common names, such as ovine rinderpest, goat plague and plague of small ruminants or Kata [12].

Clinically, the disease is characterized by high fever, severe pyrexia, ocular and nasal discharge, pneumonia, necrotizing and erosive stomatitis, ulceration of the mucous membrane and inflammation of gastro-intestinal tract leading to severe diarrhea [13, 14]. In epidemic areas, the morbidity rate of PPR is estimated from 80%-90% and mortality rate ranges from 50%-80% [15]. The disease is transmitted primarily through direct contact with infected animals through interactions with infected mucosal or fecal secretions [16]. Most recent PPR seroprevalence have been reported as 47.5%, 48.43% and 37.6% in Tigray, Oromia and Somalia regions of Ethiopia, respectively [17].

Peste des petits ruminant (PPR) is regarded as the most economically significant widespread and highly contagious viral disease of small ruminant species, particularly goats in areas where these animals are intensively reared. It continues to cause the death of millions of sheep and goats annually and is a constant threat to the livelihoods of subsistence farmers in many agro-ecological zones of Ethiopia [18, 19]. The disease is associated with high mortality and morbidity rates in naïve populations [20], significant economic losses, reduced production and productivity as well as high control costs [18, 21]. This disease is categorized as notifiable trasboundary disease by World Animal Health Organization (OIE) due to its potential for rapid spread and associated restrictions on the international trade of animals and animal products [22].

Peste des petits ruminant (PPR) causes economic losses in PPR infected countries and more than 330 million families are at risk of losing their livelihoods and food security. Additionally, small ruminants and their products are internationally traded commodities, particularly in Africa and Middle East; PPR considerably affects export earnings and creates supply shortages [23]. Considering the disease economic impact, PPR was targeted as a high priority disease for progressive control by the World Organization for Animal Health (OIE) and the Food and Agriculture Organization (FAO) to eradicate the disease at 2030. Despite the huge economic consequences and threats to trade, information on the sero-prevalence and associated risk factors of PPR infection in the study districts in particular and afar region in general is insufficient. A better understanding of its seroprevalence and associated factors would lead to improve disease control measures. Therefore, the current study was intended to estimate the seroprevalence and to assess associated factors of PPR infection in the study districts of afar region, Ethiopia.

## Results

The demographic characteristics of study population were presented in Table 1. Majority of study population, 81.51% (*n* = 313) were females while about 18.49% (*n* = 71) of them were males.

**Table 1:** The demographic characteristics of the tested animals

Variable	Category	Frequency (%)
Sex	Female	313(81.51)
	Male	71(18.49)
Species	Sheep	110(28.60)
	Goats	274(71.40)
Age	Young (6 months-1.5 year)	178(46.35)
	Adult (1.5 year < X ≤ 2.5 years)	148(38.54)
	Old $($ Y ≥ 2.5 years)	48(12.5)
Herd size	Small ($$<50 animals)$$	94(24.48)
	Medium (51-100animals)	100(26.04)
	Large ($>100 animals$)	190(48.48)
District	Asayita	179(46.61)
	Mille	206(53.64)
Total		384(100%)

### Seroprevalence of PPR infection

In the current study, out of 384 sera collected from the study population and tested using competitive ELISA (c-ELISA), 60.15% (*n* = 231/384; 95% CI of 55.15–64.95) were found to be positive for the presence of antibodies against PPR virus (PPRV) infection. The seroprevalence of PPR virus among the species level was 38.18% in sheep and 68.98% in goats in the study districts of Afar region. From the total sera tested, 23 positive samples were from males and 208 positive sera were from females with a prevalence of 32.39% (*n* = 23/71) and 66.45% (*n* = 208/313), respectively.

### Associated factors of PPR infection

Associated risk factors such as species, sex, age, herd size, district and body condition score (BCS) were assessed using structured questionnaire for every sampled herd for the occurrence of PPRV infection seroprevalence as depicted in (Table 2). In different age groups, the seroprevalence in the study population between six months to 1 year (young age group) was 72.97%, from 1 year to 2.5 years (adult age group) was 63.48% and above 2.5 years old 20.83% (old aged group). The seroprevalence of PPR infection between sex groups showed that it was 66.45% in female and 32.39% in male and it was statistically significant variation between sex groups. In the present study, the chi-squre (X^2^) analysis result indicated that among the associated risk factors with PPRV seroprevalence occurrence; species, sex, age, and herd size were found to be statistically significant as depicted (Table 2).

**Table 2:** Chi-square analysis results of associated factor of PPRV seroprevalence

Hypothesized Associated Factor		Sample size	Seropositive	X^2^ Value	*P*-value
Species	Ovine	110	42	31.0576	0.000
	Caprine	274	189		
Sex	Male	71	23	28.0093	0.000
	Female	313	208		
District	Mille	207	126	0.0953	0.758
	Asayita	177	105		
Age	Young (6 months-1.5 year)	178	113	55.5311	0.000
	Adult (1.5 < X < 2.5 year)	148	108		
	Old (Y ≥ 2.5 Year)	48	10		
BCS	Good	263	157	0.0738	0.786
	Poor	121	74		
Herd size	Small (< 50)	94	54	12.9241	0.002
	Medium(50 < X < 100	100	47		
	Large(> 100)	190	130		

Univariable logistic regression analysis was conducted to reduce the non-important hypothesized risk factors with a critical *P*-value of = 0.25. Univariate logistic regression analysis result revealed the associated risk factors that had a significant association with c-ELISA sero-positivity were species (*P* = 0.000), sex (*P* = 0.000), age group (*P* = 0.000) and herd size (*P* = 0.000). Whereas, the study areas (*P* = 0.758) and body condition score (*P* = 0.786) had not significant association with c-ELISA seropositivity of the disease as depicted (Table 3).

**Table 3:** Univariable analysis results of associated factors for PPR seroprevalence

Associated factors		Positive samples	Prevalence	*P*-value	COR (95% CI)
Species	Ovine	42	38.18%	-	Ref
	Caprine	189	68.98%	0.000	3.6 (2.27–5.71)
Sex	Males	23	32.39%	-	Ref
	Females	208	66.45%	0.000	4.1 (2.39–7.16)
Age	Young (6 m-1 year)	113	63.48%	0.000	12.96(5.99–28.04)
	Adult (1–2.5 years)	108	72.97%	0.000	8.3 (3.96–17.60)
	Old (2.5 < X < 4 years)	10	20.83%	-	Ref
BCS	Poor BCS	74	61.16%	0.786	1.06 (0.68–1.65)
	Good BCS	157	59.69%	-	-
District	Asayita	124	69.27%	0.758	0.9 (0.62–1.41)
	Mille	108	52.43%	-	Ref
Herd size	Small herd size	94	57.45%	-	Ref
	Medium herd size	100	47%	0.146	0.7 (0.37–1.16)
	Large Herd Size	190	68.42%	0.0489	1.6 (0.96–2.67)

As a rule of thumb, variables whose *p*-value less than 0.25 along with the variables of clinical importance would be selected. Accordingly, species, age, sex and herd size were found to be statistically significant variables in univariable logistic regression and were fitted to the final multivariable logistic regression model to check the real significant contribution of these associated risk factors without compounding effect on the other as depicted in (Table 4) with adjusted odds ratio (AOR) and hence, species, sex, age and herd size of animals were identified as associated factors for the occurrence of sheep and goat PPRV infection. The odds of sero-positivity in female animals were 3.9 times higher than male animals (AOR = 3.9; 95% CI = 2.09–7.39%). The odds of being caprine were 4.4 (AOR = 4.4; 95% CI = 2.57–7.65%) times more likely to be seropositive than ovine species. The odds of young and adult sheep and goats were 12.34 (AOR = 12.34; 95% CI = 5.31–28.64%) and 5.0 (AOR = 5.0; 95% CI = 2.24–11.20%) times more likely to be seropositive than old aged animals, respectively. The odds of large-sized flocks of sheep and goats were 1.92 (AOR = 1.92; 95% CI = 1.19–3.13%) times more likely to be seropositive than small-sized flocks as shown in (Table 4) which means the study population that were found in large herd size 1.92 times more likely to develop PPR virus infection as compared to animals found in small herd size (< 50 animals).

**Table 4:** Multivariable logistic regression analysis of associated risk factors and PPR seropositivity in sheep and goats

Associated risk factors		No of animals tested	Positive samples	*P*-value	AOR (95% CI)
Species	Ovine	110	42	-	Ref
	Caprine	274	189	0.000***	4.4(2.57–7.65)
Sex	Males	71	23	-	Ref
	Females	313	208	0.000***	3.90(2.09–7.39)
Age	Young (6 m-1 year)	178	113	0.000***	12.34(5.31–28.64)
	Adult (1–2.5 years)	148	108	0.000***	5.0(2.24–11.20)
	Old (2.5 < X < 4 years)	48	10	-	Ref
Herd size	Small herd size	94	54	-	Ref
	Large Herd Size	190	130	0.008*	1.9 (1.19–3.13)

## Discussion

Despite the fact that the Food and Agriculture Organization (FAO) and the World Organization for Animal Health (OIE) intend to eradicate the disease by 2030, the results of various studies revealed that an increasing seropositivity of PPR trend in sheep and goats in Ethiopia. The present study confirmed an overall seroprevalence of 60.15% of which 38.18% in sheep and 68.18% in goats were exposed to PPR viruses’ infection. This seroprevalence of PPRV infection was relatively in agreement with previous study reports such as; 64.5% from eastern Amhara region [24], 54.8% in Gambella region [25], 61.8% in Sudan [26], 55% in Nigeria [27], 55.2% in Uganda [28] and 55.95% in Saudi Arabia [29]. On the contrary, the current study result was slightly higher than previous study findings in Ethiopia such as; 36.6% study report before 10 years in Awash Fentale, afar region [30], 48.43% in Eastern Showa and Arsi Zones of Oromia Region and 43.6% in Adigudam and Chercher of Tigray [31], 22.4% in Turkey [32], 33% in India [33], 26% in Bangladish [34], 22.1% in Tanzania [35] and 34.2% in Pakistan [36]. This seroprevalence results of PPR variation in different regions as well as countries could be attributed to differences in husbandry practice within diverse geographical regions, levels of immunity, agro-ecology, diagnostic test, sampling procedures used, technical know-how of the researchers, socio-economic status of individual farmers, variation in veterinary service including vaccination program, uncontrolled animal movement and frequent contact between flocks and migration of livestock within and between countries [33].

Among the risk factors considered in the current study; species, sex, age and herd size were found to be statistically significant (*P* < 0.05). In Ethiopia, several studies indicated that goats are more severely affected by PPR virus than sheep, and they show prominent clinical symptoms while sheep only experience milder forms of the disease [37]. In the present study, the seropositivity of PPR virus infection in goats was found to be (68.98%) and 38.18% in sheep. In this case, goats were 4.4 times more likely to be seropositive to PPR infection as compared to sheep. This study is in agreement with previous epidemiological studies of [38–40], who reported a higher seroprevalence in goats than in sheep and associated it to higher fertility in goats compared to sheep. Moreover, the seroprevalence of PPR between the species showed high prevalence of 34% in goats and 24.2% in sheep which was statistically significant (*P* = 0.000) [37]. On the contrary, our study disagrees with previous studies, who reported higher seroprevalence in sheep than goats [41–43].

In this study the seroprevalence of PPR infection among sex groups was 66.45% in females and 32.39% in males. This relative seroprevalence variation was statistically significant, which means being female animals are more likely to develop PPR virus infection as compared to male animals (*P* = 0.000) and this study finding is consistent with previous studies [27, 31, 44, 45]. In the current study, the higher prevalence in females than in males may be due to physiological differences where females reveal some degree of predominance infection as a result of production and reproduction stress which makes females more prone to infection [25]. Moreover, it may also be due to livestock breeding pattern of Nepalese farmers in which females are kept longer for reproduction while most of the males are castrated and sold for meat purpose. The longer the females are kept for herd maintenance the more chances of exposure to the environment they get may result into more seroprevalence [46].

The association of age groups with PPR seroprevalence occurrence showed that age factor was found to be significantly associated risk factor (*P* = 0.000). The current study revealed the higher PPR seropositivity in adult sheep and goats (72.97%) as compared to young animals (63.48%). In this study, higher prevalence among adults followed by young animals was observed. This statement is consistent with earlier studies [47–49] that report a decreasing seroprevalence rate as age decreases, and it is due to the higher likelihood of adult animals being exposed to PPRV than younger animals. In addition, the higher seroprevalence among adults may be because long life time allowing more exposure to PPRV. On the other hand, passive immunity from dam to the young animals might have effect on the result to some extent. However, the current study result contradicts with those of other previous studies [50, 51] that showed young animals were more susceptible than adults and had a higher seroprevalence incidence than older animals.

In the present study finding, seroprevalence of PPR was also significantly affected by herd size. Herd size and seropositivity of PPR virus infection were statistically significant (*P* = 0.000). Large herd sized of sheep and goats were 1.9 times more likely to develop PPR infection as compared to those animals from small herd size keeping the other factors constant (OR = 1.9; 95% CI = 1.19–3.13%). This study result is consistent with previous reports of [52, 53]. This direct association might be an indication of the contagious nature of the disease and mode of transmission, which is attributed to crowding of animals that can facilitate the frequency of direct contact and hence escalating chances of transmission.

The only factors that were not shown to have a significant association with seropositivity to PPRV in the current study are study areas and body condition score. According to the current study's multivariate regression analysis, there was no statistically significant variation in seropositivity among the study districts. This statement is supported by previous study finding [54]. This could be probably because of PPR is persistent and evenly distributed in the study areas. These results could indicate that PPR is endemic and extensively circulating within these districts. Moreover, the study areas have more or less similar agro-ecological conditions.

## Conclusions

The present study result indicated that a higher seroprevalence of PPR virus infection that confirms the disease is circulating in Afar region. Among the associated risk factors considered in this study, species, sex, age and herd size were found to be statistically associated with the seropositivity of PPR infection. Further studies that cover the entire region and nationwide to determine seroprevalence are strongly recommended to design appropriate control and eradication strategies of PPR disease.

### Materials and methods

#### Description of study area

**Fig. 1:**
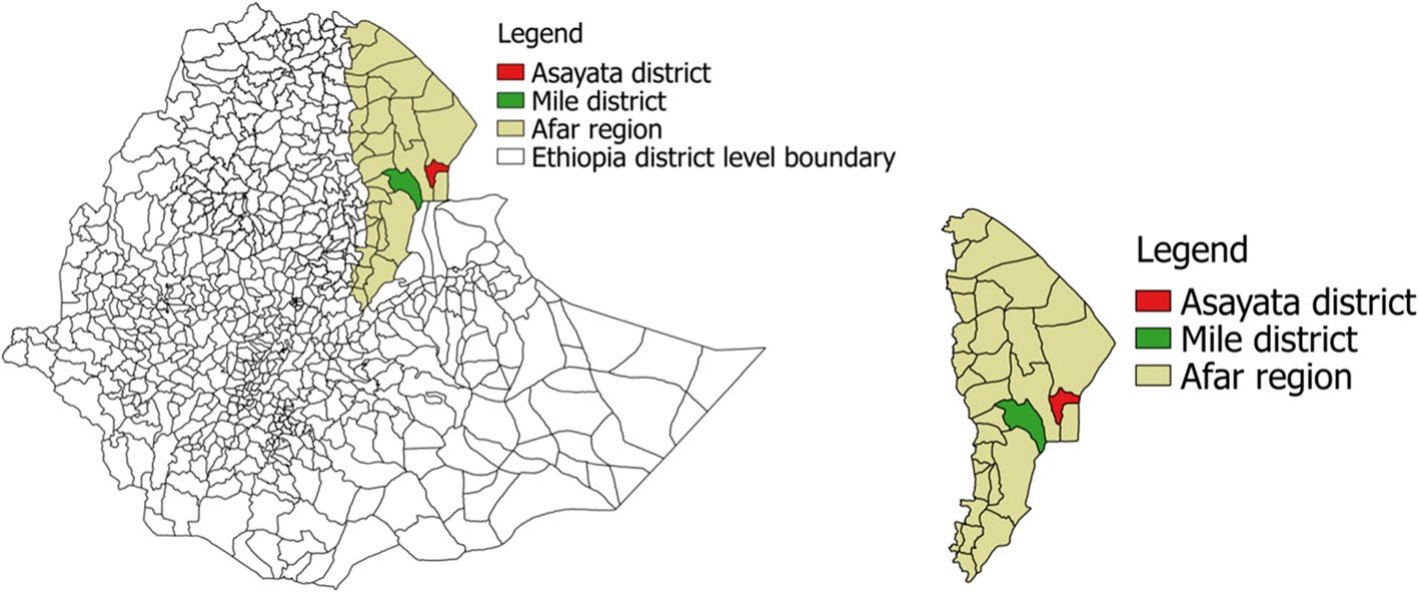
Map of Afar region indicating the study districts

The study was conducted from February to April, 2020 in two districts namely (Asayita and Mille), which are located in the administrative zone one of afar region, Ethiopia. The afar pastoral region is located in northeast of Ethiopia between 39°34’ to 42°28’E longitude and 8°49’ to 14° 30’ N latitude (Fig. 1). The region shares common international boundaries with Eritrea in the northeast and Djibouti in the east and it is characterized by an arid and semi-arid climate with low and erratic rainfall. Rainfall is bi-modal throughout the region, with a mean annual rainfall below 500 mm in the semi-arid western escarpments and decreasing to 150 mm in the arid zones to the east. The altitude of the Region ranges from 120 m below sea level in Danakil depression to 1500 m above sea level. Temperatures vary from 20 °C in higher elevations to 48 °C in lower elevations. The human population of Afar region is 1.5 million in which the majority are pastoralists who largely depend on livestock production for their livelihood. The study populations were managed under pastoral husbandry which allows high mobility of animals and these animals are usually mixed with other animal species. Sheep and goats that were kept under the extensive farming system [1].

### Study population

The study populations were sheep and goats aged greater than 6 months considered in this study. Blood samples were collected from non-vaccinated sheep and goats for the last one year. The age category of study population was classified as young (6 months to 1.5 year); Adult (1.5 < X ≤ 2.5 years) and old age (Y > 2.5 years) [55].

### Study design and sampling strategy

A cross-sectional study design with three stage sampling method was employed (districts, pastoral association (PA)/kebeles and village/herd) to reach to the sampling units as of February to April, 2020. The study districts were purposively selected based on higher study population, access to transportation, history of no vaccination for the last one year, absence of outbreak cases and willingness of pastoralists to participate in this research work. In this study, the first stage was districts, the second stage was pastoral association/kebeles and the third stage was the village/herd. A village was considered as one flock of sheep/goats (single-level clusters) that share common grazing areas and watering points. Finally, simple random sampling technique was employed to select individual animals from target population during sample collection.

### Sample size determination

Although sheep and goats are two species, they can be considered as one study population due to the management practices and the similar course of the diseases in both species. So, the sample size was determined according to the formula given by [56], using 50% expected prevalence (since there is no previous seroprevalence report of PPR infection in the study areas), 5% desired absolute precision and 95% confidence interval as below:

$$\mathrm n=\frac{\mathrm Z^2\mathrm x\;{\mathrm P}_{\exp}\left(1-{\mathrm P}_{\exp}\right)}{\mathrm d^2}$$

where: n = required sampling units.

Z = Multiplier from normal distribution at 95% Confidence interval (1.96).

P_exp_ = Estimated (expected) prevalence 50% (0.5).

(1-P) = Probability of having no disease 50% (0.5).

D = Desired absolute precision 5% (0.05).

Sampling was proportionally distributed based on the total small ruminants’ population in the study districts’ kebeles. The number of sheep and goats sampled is proportional to the herd sizes as well as within the district. Accordingly, a total of 384 sheep and goats from twenty four herds and eight kebeles or peasant associations and two districts were included in this study.

### Sample collection and transportation

Whole blood samples approximately 6-8 ml was collected from the jugular vein of non-vaccinated sheep and goats using plain 10 ml vacutainer tubes and 19 gauge sterile needles. The samples were labelled to allow identification of each animal. The associated risk factors (such as species, age, sex, herd size, body condition score and study areas) were recorded during sampling. Collected samples were kept in slant position overnight at room temperature to allow serum separation. Then, serum was decanted and aliquoted into cryovials and stored in a freezer (-20°C) at microbiology laboratory of Samara University, and transported to National Veterinary Institute (NVI) in order to detect for antibodies against natural PPR infection exposure using serological analysis. All sera samples were transported to NVI laboratory in icebox and stored at -20°C until processed.

### Laboratory analysis

#### Antibody detection against PPR infection

Serum samples were analyzed at the National veterinary Institute (NVI, Debre Zeit, Ethiopia) using a competitive ELISA kit (c-ELISA kit) according to the instructions of the manufacturer (Institute for Animal Health, Pirbright Laboratory, UK) [57]. A monoclonal antibody (MAb) based competitive Enzyme Linked Immunosorbent Assay (c-ELISA) was used for the detection of antibodies directed against the nucleoprotein of the PPR virus using approved competitive ELISA kit. Briefly, the ELISA wells were coated with purified recombinant PPR nucleoprotein (NP); the samples to be tested and the controls were added to the micro-wells. Anti-NP antibodies, if present, form an antibody-antigen complex which masks the NP epitopes. An anti-NP-peroxidase (HRP) conjugate was added to the micro-wells and incubated. It fixes to the remaining free NP epitopes, forming an antigen-conjugate-HRP complex. After washing (to eliminate the excess conjugate), the substrate solution (TMB) was added and the resulting coloration depends on the quantity of specific antibodies present in the sample. Stop solution (sulfuric acid) was added to each well in order to stop the reaction. The micro-plates were read with ELx800 Absorbance Micro-plate Reader (Biotek® Instruments, Inc. USA) with an inference filter of 450 nm and connected to a computer loaded with Gen 5TM software for automated reading and calculation of the competition percentage (S/N %) values. The OD (optical density) values of each sample were converted to S/N % by using the following formula: S/N % = [OD sample/OD NC)] X 100. The samples with S/N less than or equal 50% were considered as positive. The same procedure was used in this study to convert the OD values to percentage inhibition for PPR detection by using the following formula: PI = [100-(OD sample/OD NC)] X100. An inhibition of more than 50% was considered positive.

### Administration of questionnaire survey

There is no serological test available to differentiate animals vaccinated with PPR vaccine from animals that had recovered from a natural PPR in Ethiopia. Therefore, questionnaire was deemed to gather information regarding vaccination status of sheep and goats to aid in sampling. A structured questionnaire format was prepared to interview individual sheep and goat owners. Respondents from each district were randomly selected and interviewed to assess associated factors of PPR disease such as; species, sex, age, herd size and study areas. All necessary epidemiological information was collected on individual animal bases.

### Data management and statistical analysis

All collected data generated from field and laboratory analysis was entered in to the Microsoft excel sheet data management and analysis Window® 2007 and then it was analyzed using Stata version 14 software. Descriptive statistics was employed to quantify the results of seroprevalence of PPR antibodies. The seroprevalence of PPR virus infections was calculated as the number of PPR positive animals divide to the total population at risk of acquiring the disease [58]. The association of associated factors such as different location, species, sex, herd size and age to the results of seroprevalence of PPR infection was analyzed using Univariable and multivariable logistic regression model. A statistically significant association between variables was said to exist if the calculated *P*-value is less than 0.05 at 95% confidence interval (CI).

## Data Availability

The datasets generated and/or analyzed during the current study are not publicly available due to the confidentiality agreements made all authors, but could be available from the corresponding author on reasonable request.
